# Employer representatives’ experiences of supporting employees with cognitive impairments in a digital work environment

**DOI:** 10.3233/WOR-230690

**Published:** 2024-11-08

**Authors:** Monika Lindberg, Maria Larsson-Lund, Anna Berg Jansson, Maria Ranner

**Affiliations:** a Department of Health, Education and Technology, Division of Health, Medicine and Rehabilitation, Luleå University of Technology, Luleå, Sweden; b Department of Social Sciences, Technology and Arts, Division of Humans and Technology, Luleå University of Technology, Luleå, Sweden

**Keywords:** COVID-19, human resources, managers, multiple sclerosis, Parkinson disease, stroke, sustainable work, vocational rehabilitation

## Abstract

**BACKGROUND:**

Digital work poses cognitive demands on all employees, but the impact is greater for employees with cognitive impairments. Digitalization also has significant implications for employer representatives as they are responsible for the work environment. However, knowledge is scarce concerning employer representatives’ perspectives on identifying needs and support for employees with cognitive impairments working in a digital work environment.

**OBJECTIVE:**

To describe employer representatives’ experiences of work environment management with focus on employees with cognitive impairments working in a digital environment.

**METHODS:**

Focus group methodology was used. Six employer representatives with work environment responsibilities participated.

**RESULTS:**

One overall theme “*Mastering the interconnected processes in a transformative digital work environment*” as well as three themes “*Facilitating good digital work conditions*”, “*Identifying needs and difficulties in work tasks among employees*’ *with cognitive impairments*” and “*Pursuing knowledge and collaborations to support employees with cognitive impairments*” with subthemes were identified. The themes describe employer representatives’ challenges and efforts to identify fluctuating needs in employees with cognitive impairments and, also, to organize and reduce cognitive demands in the work environment to support them.

**CONCLUSIONS:**

Managing the challenges of an evolving digital work environment and matching individual work ability of employees with cognitive impairments in relation to cognitive demands is an ongoing process. The participants valued cooperation with employees with cognitive impairments but lacked support from expertise. The need to develop and implement a functioning support system for vocational rehabilitation to ensure a sustainable work in digital work environments is indicated.

## 1 Introduction

The continuous process of digitalization and technological development has changed the working conditions, the nature of work, and its execution and organization [1]. A digital work environment has become a natural part of the work environment and digital work is made possible by digital technologies [2]. Digital work can be described as involving the use of digital technologies most of the working hours that also can enable flexibility in terms of when and where were work is conducted [3]. In the aftermath of the COVID-19 pandemic, many employees have been afforded the opportunity to work both from home and on site in the physical workplace. This hybrid way of working thus seems to be made permanent in many sectors [4, 5]. However, these flexible and digital work arrangements may pose both challenges and opportunities for employees. This approach requires planning and structuring of the workday e.g., deciding where and when to work as well as prioritizing between work tasks [6–8]. Digitalization also requires more multitasking and implies an increased information load as well as a higher work pace [8–10].

The digitalization and the integration of hybrid work also have significant implications for employers’ leadership and responsibility for the work environment within the digital workplace [11]. Unfortunately, there are few organizational policies or guidelines on hybrid work and how to ensure the health of employees working from home [4, 12]. Digitalization imposes cognitive demands on all employees within an organization [8–10]. However, these demands are presumably more demanding for people experiencing cognitive impairments related to a disease or an injury [13] and there is a need to recognize those and their needs, which is rarely done [14]. Consequently, the literature indicates that the work environment management must be adapted to meet the specific needs of employees with cognitive impairments, but knowledge of how to do this is scarce.

Cognitive impairments are common consequences related to stroke, multiple sclerosis (MS), Parkinson’s disease (PD) or post COVID-19 syndrome (PCS) [15–20]. Cognitive impairments, including perceived difficulties with concentration, attention, and memory, as well as heightened sensitivity to distressing stimuli and reduced tolerance for stress [16, 19–21] as well as fatigue [22–25] are associated with these disorders. These impairments may be manifested e.g., in difficulties working in shared offices due to sensitivity to disturbances and noise, difficulties performing multiple work task in parallel or an increased need to take brakes during the workday [14]. Cognitive impairments are often unnoticed by others during work-related activities. This can be attributed to the subjective nature of the experience and the challenges faced by the affected person in comprehending and articulating these difficulties [26]. Furthermore, objectively measuring these impairments in work life presents a significant challenge [14]. The lack of visibility of cognitive impairments can lead employers and colleagues to be unaware of the difficulties, with the risk of their needs being overlooked [26]. Having stroke, MS, PD or PCS may impact the abilities and possibilities to work and many people are of working age when affected [27–30]. Consequently, it is important to understand whether the needs of employees with cognitive impairments are met.

A person’s ability to work is influenced by the interaction between the person, the work and the environment, including supporting and hindering elements in them. Thus, the work ability is dependent on this interaction and not only on the person’s state of health. This implies that the ability to work can be improved by changing the interaction e.g., by reducing cognitive demands in the environment for people with cognitive impairments [31]. Physical, psychological, cognitive and social aspects of work are all part of an accessible work environment. In Sweden, the employer has legal obligations with responsibility for systematically creating a health-promoting work environment [32, 33] and work adaptations for individual employees [34]. In addition, the health care service is responsible for the medical rehabilitation and the Social Insurance Agency is responsible for assessing the person’s ability to work [32]. Support from employers has a crucial role in facilitating a sustained employment [14, 35, 36]. However, the lack of experience and knowledge about cognitive impairments and their impact can be a challenge in the endeavor to work as a supportive employer [36] but cooperation with the employee can make it easier to manage, continue and return to work for employees [37, 38].

Research regarding employers’ perspectives on supporting employees with cognitive impairments related to neurological disorders when working in a digital work environment is limited. More knowledge is needed regarding how employers manage issues related to occupational health in digital work environments, as well as the identification and support of employees with cognitive impairments. Consequently, this knowledge can facilitate the development of adequate interventions and support systems aimed to retaining skilled personnel with cognitive impairments at work to promote an inclusive and sustainable work life for all. The aim of this study was to describe employer representatives’ experiences of work environment management with focus on employees with cognitive impairments related to stroke, MS, PD and PCS working in a digital environment.

## 2 Methods

### 2.1 Design

A qualitative design with focus group discussions was used to explore participants’ experience of managing the work environment, focusing on employees with cognitive impairments. The method was selected due to its stimulating dialogue and interaction between participants allowing them to draw on and develop each other’s thoughts to create a collective understanding of this new area of research [39, 40].

### 2.2 Participants

Six people participated in two focus groups. Snowball sampling [41] was used for recruitment to reach a purposeful sample with varied experiences. The process began by recruiting participants through our networks. Managers or human resources (HR) personnel (hereafter referred to as employer representatives) with work environment responsibilities and experience of supporting employees with cognitive impairments related to stroke, MS, PD or PCS working in Sweden were contacted and invited to participate. Their employees needed to work with digital technologies, such as computers, smartphones, various software programs required for the work for at least half of their working time. Potential participants were requested to suggest other people with the needed experience for participation. However, this recruitment method did not yield the expected results, and an extended recruitment procedure was therefore undertaken. The internet was utilized to identify employer representatives who were e-mailed with information about the study and a question to participate. If no response was received within a week, a reminder e-mail was sent. The ambition was to obtain a purposeful sample that was homogeneous and heterogeneous to achieve a broad representation of the target group and facilitate discussion [39, 40, 42]. Over a hundred requests were sent through both methods of recruitment but many were not replied to despite reminders. Six people representing a sample with a purposeful variation responded positively after a five-month recruitment process. Homogeneity was achieved concerning profession and experience of supporting employees with cognitive impairments related to stroke, MS, PD or PCS. Heterogeneity was achieved by including employer representatives from small and larger companies and business areas in the public, private and nonprofit sectors. Additionally, gender, age and years of work experience varied among the participants (Table 1).

**Table 1:** Characteristics of the participants, *N* = 6

Background variables
*Gender*
Female	5
Male	1
*Age*
Median (range)	47 (33–54)
*Profession*
Manager	4
HR personnel	1
Manager and HR personnel	1
*Sector*
Private (industry, and civil engineering)	2
Public (municipal administration, Swedish authority administration, and call-centre)	3
Nonprofit (education)	1
*Number of employees*
Median (range)	24 (8–300)
*Years of professional experience*
Median (range)	25 (13–32)
*Years of experience as manager or HR-personnel*
Median (range)	11,5 (5–26)

### 2.3 Data collection and procedure

Data were collected in two focus group discussions with three participants in each in November 2022 and March 2023. Prior engaging in the focus group discussion, the moderator (ML) called the participants and provided them with details regarding the study and the nature of focus group discussions, they were also given the opportunity to ask questions. During this phone call demographic data were also collected. Participants were given oral and written information about participation being voluntary and the possibility of withdrawing at any time without explaining the reason [41]. They were also assured that their identities and the names of their workplaces would be kept confidential in the study. They were informed not to name the employees with cognitive impairments they were referring to, in order to keep the employees anonymous. The participants gave their written informed consent prior taking part in the focus group discussions.

The focus groups were moderated by the first author (ML) and co-moderated by the last author (MR). The discussions started with the moderator describing the aim of the study, the structure for the focus group and stressing out that the participants were the experts of the topic and the importance of them sharing their thoughts and experiences freely. Thereafter participants briefly introduced themselves and their workplaces. The focus groups discussed the following topics: *challenges and opportunities a digital work environment may pose, opportunities and challenges in work environment management, experiences of supporting employees with cognitive impairments working in a digital environment and how to prevent work-related ill health related to the digital work environment and cognitive demands*. The moderator was responsible for that everyone in the focus group had the opportunity to make their voices heard, stimulated the interaction and put effort in listening without interrupting the discussion. The co-moderator took fieldnotes on the interactions and nonverbal situations and summarized the discussion [39, 43]. One focus group was conducted at the university in a secluded room and lasted for ≈115 minutes. The other was conducted remotely using video conferencing technology, given the geographical spread of participants across different regions of Sweden and lasted for ≈90 minutes. Both focus groups were audio recorded and transcribed verbatim. Following each focus group discussion, the moderator and co-moderator summed up their thoughts, impressions, fieldnotes and interactions in the group. This summary was recorded and included in the data analysis.

### 2.4 Data analysis

The analysis was carried out in a stepwise procedure according to focus group methodology [39, 40]. All text was kept in Swedish as far as possible in the analysis procedure to stay close to the raw data, to not lose its content and meaning. In the first step of the analysis, the first (ML) and last author (MR) listened to the audiotaped discussions, read the transcripts and the fieldnotes several times, guided by the aim, to become familiar with the material and understand it in its context. Notes were taken on preliminary interpretations and were jointly discussed. In the next step, the data were sorted by the first author, guided by preliminary interpretations and formed preliminary themes. In the third step, the sorted data were condensed to describe the content of the focus group discussions. The first and last authors discussed the meanings of the condensations and compared them to the preliminary themes and subthemes that had arisen. Example of preliminary themes at this stage: *Detecting and identifying needs related to cognition*” *and* “*Managing the digital work environment*”. These were further developed by discussions between all authors based on data. In this step, the two authors (MLL and ABJ) asked questions about the evolving themes based on the content of the transcripts. Finally, in the last step all authors refined the themes and subthemes and reached an understanding of the underlying meaning of the common understanding of the focus group discussions in an overall theme.

## 3 Results

The analysis resulted in the overall theme “*Mastering the interconnected processes in a transformative digital work environment*”. The theme describes participants’ collective experiences addressing the challenges identifying the abilities and needs of employees in digital work environments, with a specific emphasis on employees with cognitive impairments related to cognitive demands. Added to this were their efforts to organize work in response to the constantly changing conditions arising from the implementation of new technologies and the variations in abilities of their employees with cognitive impairments. As new risks and needs constantly arose in this interaction, it became evident that managing the work environment was an ongoing process that needed continuous mastery by employer representatives. However, participants perceived a lack of knowledge and sought collaborations with experts, which was lacking. Consequently, they felt alone and abandoned, emphasizing the need to establish a functioning support system to facilitate work environment management and vocational rehabilitation to ensure a sustainable digital work life for employees with cognitive impairments (Table 2).

**Table 2:** Results by overall theme, themes and subthemes

Overall theme	
Mastering the interconnected processes in a transformative digital work environment	
Themes	Subthemes
Facilitating good digital work conditions	•Organizing the work with regard to digital technologies in the workplace
	•Managing the ever-changing demands of digital work
Identifying needs and difficulties in work tasks among employees with cognitive impairments	•Building and maintaining a relationship is an ongoing process
	•Dealing with subtle and unspoken difficulties
	•Organizing the work environment according to individual needs
Pursuing knowledge and collaboration to support employees with cognitive impairments	•Using own knowledge and seeking support from professionals and personal networks
	•Seeking but not receiving guidance from other expertise

### 3.1 Facilitating good digital work conditions

This theme and two subthemes describe the challenges of organizing and managing a digital workplace to meet the needs of all employees, regardless of cognitive impairments. The digital environment posed cognitive demands and participants described various measures to limit those as far as possible. Having good digital working conditions was described as decisive when supporting employees with cognitive impairments.

#### 3.1.1 Organizing the work with regard to digital technologies in the workplace

The participants experienced that work tasks where digital technology was integrated were constantly evolving and changing and presented new risks and demands for most employees but foremost for their employees with cognitive impairments. This meant that the employees constantly needed to learn new, be updated and solve problems that arose in a digital work environment. Since the participants were responsible for the work environment, they assumed the role of advocates for their employees. Additionally, they acted as gatekeepers as they had the mandate to question the necessity of, e.g., new software programs or digital technologies, as discussed in focus group two:

Participant (P)2: We managers are usually told when a new [digital] tool is to be launched, I’m usually a bit questioning. What is the purpose? Will it be less work for the employees, or will it be another tool that makes you spend another half hour extra every week just to use the tool? A little questioning there, but at the same time critically examining, trying not to burden employees with something that only comes from above [from the management].

P3: We also make sure that if we are going to launch a new digital tool, there are test pilots. I am one of our managers who wants to get involved in this for the same reason as you [p.2]. Do we truly focus on everyone? Will this work? As we do not have the money to test a lot of different things, we need to limit ourselves. What do we need? You have to cover all kinds of people with different conditions who will use this [digital tool].

As a way to limit the cognitive demands on employees, participants were restrictive about introducing or burdening them with new technologies or systems that were not necessary in their daily work. However, when new updates or systems were to be introduced, participants tried to facilitate by informing, preparing and organizing in advance. They addressed the importance of adapting information before sharing it, particularly for employees with cognitive impairments. For example, information was conveyed through written text, videos or speech so that everyone could absorb it in the best possible way, thus trying to reduce the cognitive demands that could otherwise easily arise.

Digital technologies enabled many employees to work from home and stay connected after working hours. Participants stressed the importance of establishing organizational boundaries collectively with employees. As participants had work environment responsibility, they tried to be role models by setting boundaries between work and other everyday activities. Not sending e-mails after work hours and turning off the phone during free time were ways to set boundaries and thus set a good example. By doing so, participants communicated that employees were not expected to engage in work-related tasks or be accessible beyond their designated work hours either. The importance of organizational boundaries as illustrated by focus group one:

P1: It can also be some kind of organizational framework. We do not respond to emails at 4.35 am.

P2: Exactly

P1: We do not send emails at 8.00 pm and somebody still replies. If you want to send it at that time, you can set it to be delivered at 8.00 am the next day. That, triggers people who may have difficulties setting limits.

P3: Yes. I think it is also important as a manager not to send too many e-mails outside working hours or start trying to call someone, you set a good example.

The participants also discussed the importance of initiating breaks and facilitating in-person meetings with employees. This would enhance the social interaction and break the monotonous work by the computer, as digital technology had also transformed the way of social interaction at work.

#### 3.1.2 Managing the ever-changing demands of digital work

Participants discussed the risks and demands caused by digital technology and the hybrid way of working that affected all employees and specifically employees with cognitive impairments. By being active, present, available and a part of the employees’ everyday work life made it easier to observe changes in their abilities and capacities during the workday. Creating and having a personal relationship with each employee made participants aware of each employee’s cognitive ability and capacity which often varied for those with cognitive impairments. The participants shared common experiences concerning employees working from home and highlighted the increased difficulty in providing support to employees when encountering task-related difficulties. The participants also expressed that it was difficult to make an accurate assessment of employees’ workload through virtual interactions. Having employees work from home was discussed as more demanding as it forced participants to be more attentive to possible changes in employees’ behaviour and work performance. Their experience was that employees with cognitive impairments needed more structure and support in their daily work and it was challenging to provide this when working from home. In addition, participants had become aware that employees’ homes were now a part of the work environment and thus a work environment responsibility for them, as illustrated by focus group two:

P3: During the pandemic we did physical safety inspection rounds digitally, even at each other’s homes. You actually got an overview of what it looked like at home and found lots of things that we have corrected. So, I think that regular safety rounds, also dialogue with the employees, have given us several good questions about health and linked to the digital transformation that we are in. What kind of support do you need and how far have you come in various programmes and tools you need to be involved in?

Moderator: Is the cognitive included or is it mostly focused on the physical?

D3: No, mainly the cognitive, I would say.

P2: That is a great idea, I will also do that, if it's okay? I will steal it straight, to do safety inspection rounds at home as well. As an employer you still have the responsibility regardless of where they sit and work, you have the work environment responsibility.

P3: Yes, exactly. That is what I used as an argument too, that you cannot work at home if we cannot talk about your work environment, how it is at home.

### 3.2 Identifying needs and difficulties in work tasks among employees with cognitive impairments

This theme and three subthemes illustrate discussions about the process of identifying employees’ cognitive impairments and needs. In addition, the challenges of identifying them and organizing work accordingly.

#### 3.2.1 Building and maintaining a relationship is an ongoing process

Cooperation with employees with cognitive impairments was described as essential, because without it, supporting their needs was difficult. Participants discussed the importance of a leadership style that was open, communicative and interactive, to create a trust among employees. This style of leadership usually led to a trustful relationship where the employees felt confident in being open and expressing their cognitive impairments and need for support at work. However, cooperating and building an open and trustful relationship was not an easy fix, rather it demanded a process over time as illustrated by focus group two:

P1: If it has been a Parkinson’s diagnosis, of course you have had a lot of conversations. They have informed you, provided medical certificates, all the rehab and everything. For instance, dementia is something that at least I have been involved in discovering myself without the person in question wanting me to know about it. The hardest is when the employer has to pay attention to the person in question, and when it doesn't really work. So, you notice that the job is not done, you must double-check the job and then try to talk, involve the occupational health care, call relatives.

P3: Yes, exactly. I have the same experience when I summarize my entire work life as a manager. However, when recruiting new staff, we have had the opportunity to ask questions: Is there anything you want to tell me that you think is important for me to know? When it comes to post COVID-19, I have been engaged with them throughout their process concerning medical care and everything. All the symptoms and everything that has happened, so I have been very, very close.

P2: For me, it’s about the same. I have been involved as a manager only in the post COVID-19 case. On the other hand, I had a colleague who has been diagnosed with Parkinson’s disease. But it was discovered at a fairly early stage that there was a deterioration in work ability and the person was open about it himself.

#### 3.2.2 Dealing with subtle and unspoken difficulties

The participants’ experiences revealed that it was challenging to identify employees’ cognitive impairments or needs if they were not expressed. This meant that participants had to find other ways and methods to identify these. Two main approaches to detect employees’ difficulties and needs were described. One was being attentive to repeated sick leaves and dealing with sick leave certificates Additionally, some employees with cognitive impairments were referred to the occupational health care, which could then share information about the employee that the employee themself had not disclosed. The other way to identify difficulties related to cognition was by, e.g., following the employee’s production targets and statistics as well as how and when work tasks were completed. By being attentive and following targets and statistics, participants quickly got indications if difficulties arose and could more easily identify them, as illustrated by focus group one:

P2: They have usually become ill, or something has happened, and been away for a long time. Then, you had to work to bring them back.

P1: You handle sick leave certificates, but in our case, the two I’m thinking of in this context. They have even been in the newspaper, so they have been very open, so to say.

P2: Well, so have mine.

P1: It was like a hype around COVID-19 when it occurred. It was fun that you wanted to disclose it. There have not been any oddities.

P2: We are 65 employees. We have a person where I have seen that there is something. It doesn't really work as it should if you look at statistics. They have a lot of sick leave instead, so I send it to the occupational health care. And they have had conversations and have found out a lot of information that maybe the employees do not want to tell their manager.

#### 3.2.3 Organizing the work environment according to individual needs

An inclusive work life where employees could work despite having cognitive impairments that affected their ability to work in a digital environment was described as important. Many employees with cognitive impairments often had extensive experiences and skills that were hard to replace. Nevertheless, their abilities could vary during a workday and between days. Participants similarly realized that the employees’ work ability was also affected by their other activities outside of work and described difficulties of taking this into account in assessments. It needed participants to adopt a rehabilitating approach and develop supportive workplace adaptations that aligned with employees’ abilities to facilitate work. Identifying each employee’s needs of support to come up with adaptations was described as trial and error, as both participants and employees had insufficient knowledge about potential solutions that could work when having cognitive impairments. Organizing work and offering a flexible work life that included flexible working hours, opportunities to work from home, easier or adapted work tasks, less time working at the screen as well as prioritizing tasks and limiting cognitive demands were measures used as discussed in focus group one:

P2: We do quite a lot of adaptations, try to do as much as we can when someone has suffered a stroke or something. Right now, we have quite a few who have post COVID-19. Those who cannot be at work at all, who have to lie down in bed to be able to work. We have tried to adapt based on that because they do not want to be on sick leave. They can work but cannot work if they have to sit in the office. We do a lot of adaptations, like split shifts. Some people work the morning in the office, they have a break for two or three hours and then they work the last two hours from home. They can go home and sleep and rest because they need it in the middle of the day. These kind of solutions have been made. They have tried different pace of work and so on, we are very, very flexible as far as possible.

P3: But I think it’s a challenge when you have it the way we have. There are such very specific tasks, professions, and not so many opportunities to adapt the task.

P2: No, we do not have that either.

P3: This is how it has to be done. What can we do to make it work for the individual and for us at the office? We have had, just a long, long journey, in dialogue with this person, that maybe you can’t handle full-time? And then down to part-time, and when it doesn’t work in the office, [it has to] be at home. You constantly try to adapt as far as possible.

Nevertheless, a digitalized work environment no longer offered many easier work tasks for those with cognitive impairments. Shared experience showed that it was easier to adapt for physical disabilities, which were static, than for cognitive impairments which were dynamic, because participants had more experience with physical adaptations. They also reflected that adapting the environment costs little compared to losing employees with experience and skills and recruiting new employees. However, making adjustments involved difficult considerations in relation to other employees to avoid increasing their workload or negatively affecting their work situation.

### 3.3 Pursuing knowledge and collaboration to support employees with cognitive impairments

This theme and two subthemes describe the participants’ search for where and how to obtain knowledge, guidance and support when supporting employees with cognitive impairments in a digital work environment. Their own experience, knowledge and personal network were seen as resources. Support from or collaboration with other professionals was insufficient and left them feeling alone and unsupported.

#### 3.3.1 Using own knowledge and seeking support from professionals and personal networks

The knowledge and experience accumulated by the participants during their long work lives was seen as a resource in supporting employees with cognitive impairments. However, the participants described a continuous need for more knowledge to organize work in relation to employee’s needs. They experienced it as their own responsibility to improve their own knowledge and did it by, e.g., attending topical lectures and keeping updated with current reports, guidelines and research. Nevertheless, this was sometimes not enough, and the collective experience showed that it was necessary to have a personal network of support. This network consisted of, e.g., management team colleagues, fellow managers, other colleagues and family and friends and provided knowledge and guidance as illustrated in focus group two:

D1: I probably turn primarily to my management team, where I have both colleagues and a great boss. I think I can solve quite a lot and get support. There is always someone who has been through something and can give advice and opinions and so on.

D3: I also have private [support] and could contact friends, so that would also work.

D2: Yes, it would. I’m also lucky that I have an expert at home who can help. It's also important that you have colleagues, especially managerial colleagues who have roughly the same situation. Not all of them, of course, fortunately, but some who have had the same issues and they have tried to solve it together and share experiences as well.

Additionally, participants described receiving guidance and support from policies, laws and regulations. They discussed difficulties when employees with cognitive impairments were unable to perform the work although adaptations of workplace, work hours or work tasks were in place. In that situation receiving support from laws and policies was described as vital. However, laws and policies could also be obstacles as they made it difficult to dismiss employees who were still unable to perform work despite various measures taken.

#### 3.3.2 Seeking but not receiving guidance from other expertise

Participants discussed the challenges of organizing work and supporting employees with cognitive impairments without having sufficient knowledge of how these affect the person, their digital work or other activities in everyday life. They addressed the challenges of being responsible for systematic work environment management, which included digital technologies, without having the right competences. The participants experienced that various occupational health professionals where they were supposed to receive support from had insufficient knowledge about cognitive impairments and their impact on work and everyday life. This meant that requested support, guidance or assistance was not provided, and participants lacked supportive expertise and procedures to address the difficulties. The mutual impression was that support offered by occupational health care mostly focused on medical check-ups, physical impairments and physical ergonomics but hardly never on cognitive impairments or cognitive ergonomics.

Discussions have also focused on how cooperation with health care, occupational health care and social security in the rehabilitation process of employees has deteriorated in general in recent years. Participants sensed being left alone with the employee to solve problems that should also include other stakeholders and professionals with more expertise. The lack of support left the participants alone in vulnerable and challenging situations, as discussed in focus group one:

P2: I have two who have suffered a stroke. It has been like a proper rehab, which is what happens. I think it has become worse. Earlier you had rehabilitation meetings, social security agency would always be involved, the health care would be involved, our doctor maybe would be involved and then maybe I would be, also HR or a manager. Now it is like, if you get hold of social security agency ...  

P1: Then you get to buy cake if you succeed.

P2: Yes, really. You want to have all stakeholders around this person and sit down and decide what is best. Now you cannot always reach them, they are not involved in the same way. I don’t know if it’s because they have moved the boundaries when it comes to weighing the employees to the labour market. But I feel that it’s not the same as it was 10 years ago. It feels like there are more regulations for the employer now.

## 4 Discussion

The main finding of this study implies that managing the demands of a dynamic digital work environment to match individual work ability of employees with cognitive impairments is a challenging ongoing process that constantly needs to be mastered. This was addressed as important to promote an inclusive work life where all employees were seen as a resource and where employees could continue to work and fulfil their work commitments despite having cognitive impairments. However, to reach this, participants’ collective experiences revealed a need to develop and implement a functioning support system for work environment management and vocational rehabilitation in relation to cognitive demands and cognitive impairments. Nevertheless, when considering the transferability of the findings it is necessary to acknowledge that this study had a small sample size. Still, it is noteworthy that the findings offer an insight into the complexities associated with managing and organizing the digital work environment for employees with cognitive impairments, which to the best of our knowledge has not been presented in previous research.

The findings indicate that the digital environment is ever-changing, and new technologies are constantly being implemented in organizations. It is evident in this study that the fast and unpredictable changes were cognitively demanding especially for employees with cognitive impairments. This meant that the employer representatives continuously needed to reorganize work by adapting it to their employee’s needs. This reflect how the work ability in employees with cognitive impairments can be supported and increased by reducing the cognitive demands in the environments and, thereby altering the interaction between the person, the work, and the environment [31]. These findings are also in line with previous research [44] describing paradoxical tensions caused by digital technologies as managers lead their teams in these ongoing processes even though they themselves may be affected. The findings also show that making adaptations and organizing the digital work environment to be less cognitively demanding and more brain friendly was difficult when lacking knowledge about cognitive impairments. The challenge of supporting employees with cognitive impairment without having the right knowledge can be overcome through establishing collaboration with professionals such as occupational therapists. The findings indicate that competence in individually evaluating the impact of various cognitive impairments and how these can be reduced by self-management strategies and adjustments in work is needed. This is in line with research highlighting the need of including professionals with expertise in rehabilitation to facilitate a supportive work environment [45, 46].

In this study, it became visible that managing and organizing work in a digital workplace demanded experience, knowledge and collaboration. Participants experienced having insufficient knowledge regarding cognitive impairments and their impact on work and other activities in everyday life, yet gaining more knowledge was their own responsibility. Learning and gaining knowledge by oneself are also exemplified in research concerning the challenges of leading the digital workplace [11]. The findings imply that collaboration with stakeholders or other professionals was deficient, and participants lacked support from expertise. This is also described in previous research [36] showing the lack of cooperation with stakeholders when supporting employees in returning to work. The development of collaboration between stakeholders must therefore take place at many levels of an organization related to a digital work environment to refine the needed support. The development and implementation of a functioning support system for vocational rehabilitation including professions with expertise in assessing and facilitating work ability in relation to cognitive demands such as occupational therapists is suggested. Based on the results, it seems important to assess how the cognitive demands in a person’s other daily activities outside work influence the work ability to promote a sustainable work life. This is line with occupational therapy theory [31] where work is considered as one activity in a person’s life, alongside many others as household chores and leisure activities that together influence health and work ability. Thus, the distribution of work in relation to all other activities in a person’s everyday life influence occupational balance [47] and is important to consider in supporting a sustainable work life. However, this goes beyond the responsibility of work environment management and underscores the importance of cooperating with professionals with such responsibility and competence in the vocational process. Ultimately, by cooperating and developing the support system to include professions with needed expertise employer representatives could be better prepared to facilitate a sustainable work life for employees with cognitive impairments.

This study visualizes the sensitive, complex and ongoing process of identifying employees’ needs and difficulties at work related to cognition. People experiencing cognitive impairments may be reluctant to disclose their condition, owing to its sensitive nature and the potential consequences of being stigmatized. The findings imply that the process of identifying employees’ needs required cooperation between the employer representative and the employee over time, based on a trusting relationship which concurs with previous research [37, 38]. In addition, involving and collaborating with family members can provide valuable insights into the needs of employees with cognitive impairments [38]. Research also suggests that a trustful relationship and good collaboration are factors for successfully supporting employees [35, 48] which is a substantial finding also in this study. Early identification of cognitive impairments and needs among employees with stroke, MS, PD and PCS as well as supporting their needs is important. In particular as previous research [49, 50] shows that cognitive impairments predict sickness absence and permanent work disability in cognitively demanding occupations. A trustful relationship and cooperation with the employee as well as early identification seem to be cornerstones for achieving an inclusive and sustainable work life for employees with cognitive impairments. However, there is an ongoing debate about how work life is being challenged and changed by new ways of organizing work. New ways of organizing work, such as temporary employment contracts, project employment and temporary agency arrangements, challenge the established norm of full-time employment with a single employer [51]. These ways of organizing work life may influence the conditions for building and maintaining a trusting relationship and cooperation between employer representatives and employees [52]. Thus, this might negatively impact the possibilities to work and have a sustainable work life for employees with cognitive impairments. This could lead to restricted opportunities to engage in work which may result in occupational injustice, leading to exclusion and alienation [53].

The efforts of organizing the work environment and offering work adaptations to retain employees with cognitive impairments at work are apparent in this study. Nevertheless, doing this was in accordance with our previous research [14, 54] difficult as cognitive impairments are unpredictable and fluctuate in both degree and severity and are often influenced by various factors both inside and outside of work. Furthermore, the findings showed that offering flexible ways of working was a means to retain competent staff, and the demand for workplace flexibility is also presented in recent research [12]. Despite the benefits associated with a flexible work life, there is also a contrasting perspective. Problems may arise when work encroaches upon nonworking hours resulting in a borderless work life wherein employees exceed their designated working hours. Furthermore, this study sheds light on the challenges faced by employer representatives in relation to flexible work arrangements. The challenges of flexible ways of working are also highlighted in research [4] implying challenges for employer representatives, employees and the organization in terms of both social and environmental aspects. When work environment responsibilities have been extended to the home, a policy outlining the conditions for working from home could reduce the challenges for those responsible for the work environment. As found in research [7] as well as in this study, matching employees’ individual needs and requirements with the cognitive demands of work is imperative for maintaining their contribution in the workforce. This study revealed that employer representatives sometimes experienced difficulties matching tasks to employees’ level of cognition as digital technology had replaced many easier work tasks. Current research [55] describes same challenges when adapting work tasks to match the employee’s capacity and still be stimulating. Employer representatives encounter many challenges when trying to organize work to be brain friendly and to retain experienced and skilled staff, as less cognitively demanding tasks become rarer. The findings revealed that adapting work according to employees’ needs was not performed only once but was an ongoing process. It needed a continuous interaction between employer representatives and employees as both the cognitive demands and employees’ cognitive abilities varied.

### 4.1 Methodological considerations

Despite the extensive distribution of over one hundred invitations during the recruitment phase only six participants were enrolled in this study. This could be seen as a limitation as the two focus groups consisted of only three participants each, while the literature describes an ideal focus group consisting of four to eight people [39]. Focus groups can, however, be conducted with a limited number of participants (mini focus group) comprising two to five participants, when composed of people with high level of expertise [56] as it was in this study. Groups with fewer participants enable everybody to take more part in the discussion [57]. On the other side, a small group with few participants may impact the group dynamic and limit the range of perspectives presented in the discussion, which may have impacted the results of the study. However, the two discussions provided rich information, despite limited number of participants. Several factors may have influenced the restricted number of participants, e.g., the lack of experience among employer representatives due to the invisibility of cognitive impairments or the early retirement of employees with cognitive impairments. Moreover, an uncertainty about whether an employer representative is doing the right thing when lacking knowledge about cognitive impairments and their consequences in digital work, may have deterred potential participants from engaging in the study. Despite efforts to increase male representation, the study ultimately included one male participant, which can be considered a limitation.

Another limitation is that the data collection was carried out in two different ways. Given the geographical spread of participants across different regions in Sweden, video conferencing enabled participants with expertise in the topic to take part. All participants were used to videoconferencing and there were no technical failures during the focus group discussion. By preparing and having well-functioning technology, digital focus groups via video conferences are an alternative way to collect data [39, 58]. To facilitate transferability [41] the sampling and characteristics of participants, data collection and analysis were accounted for. To strengthen the trustworthiness [41] the data, analysis, and results were discussed among all authors. The authors were involved in the steps of the analysis in different ways and had various experiences and preunderstandings [41, 59] in using qualitative methods and from working in different research fields. This leading to different perspectives and a broader view of the subject which strengthened the analysis.

## 5 Conclusions

The findings provide an insight in that employer representatives can experience a variety of challenges when managing the demands of an evolving digital work environment. It was described as an ongoing process where taking single actions was not enough because the cognitive demands and the cognitive capacity of the employees with cognitive impairments were constantly changing. Employer representatives valued the support given from their personal networks. Nevertheless, they requested more collaboration and support from expertise included in a well-functioning support system, which was lacking. The employer representatives often felt alone supporting employees with cognitive impairments and expressed that the support provided was insufficient and that there should be more to offer. Therefore, it is valuable to develop the support system and vocational rehabilitation to include professionals, such as occupational therapists, with expertise in how cognitive impairments affect work and other activities of daily living, as well as knowledge of how to assess work ability. The knowledge derived from this research is relevant for stakeholders and authorities but needs further exploration. However, the knowledge gap remains, calling for future research focusing on strategies in work environment management to facilitate employees with cognitive impairments to remain at work and have a sustainable work life.
